# The use of one-stage meta-analytic method based on individual participant data for binary adverse events under the rule of three: a simulation study

**DOI:** 10.7717/peerj.6295

**Published:** 2019-01-23

**Authors:** Liang-Liang Cheng, Ke Ju, Rui-Lie Cai, Chang Xu

**Affiliations:** 1 West China School of Public Health, Sichuan University, Chengdu, China; 2 West China Research Center for Rural Health Development, Sichuan University, Chengdu, China; 3 Chinese Evidence Based Medicine Center, West China Hospital of Sichuan University, Chengdu, China

**Keywords:** IPD meta-analysis, Evidence synthesis methods, Multilevel logistic, Rule of three, Binary rare adverse event

## Abstract

**Objective:**

In evidence synthesis practice, dealing with binary rare adverse events (AEs) is a challenging problem. The pooled estimates for rare AEs through traditional inverse variance (IV), Mantel-Haenszel (MH), and Yusuf-Peto (Peto) methods are suboptimal, as the biases tend to be large. We proposed the “one-stage” approach based on multilevel variance component logistic regression (MVCL) to handle this problem.

**Methods:**

We used simulations to generate trials of individual participant data (IPD) with a series of predefined parameters. We compared the performance of the MVCL “one-stage” approach and the five classical methods (fixed/random effect IV, fixed/random effect MH, and Peto) for rare binary AEs under different scenarios, which included different sample size setting rules, effect sizes, between-study heterogeneity, and numbers of studies in each meta-analysis. The percentage bias, mean square error (MSE), coverage probability, and average width of the 95% confidence intervals were used as performance indicators.

**Results:**

We set 52 scenarios and each scenario was simulated 1,000 times. Under the rule of three (a sample size setting rule to ensure a 95% chance of detecting at least one AE case), the MVCL “one-stage” IPD method had the lowest percentage bias in most of the situations and the bias remained at a very low level (<10%), when compared to IV, MH, and Peto methods. In addition, the MVCL “one-stage” IPD method generally had the lowest MSE and the narrowest average width of 95% confidence intervals. However, it did not show better coverage probability over the other five methods.

**Conclusions:**

The MVCL “one-stage” IPD meta-analysis is a useful method to handle binary rare events and superior compared to traditional methods under the rule of three. Further meta-analyses may take account of the “one-stage” IPD method for pooling rare event data.

## Introduction

Meta-analysis is a statistical method that quantitatively combines the findings of studies about similar questions with a certain “averaging” approach (Dersimonian & Laird, 1986). This analytical procedure has become the critical basis of healthcare decisions, because it maximizes the strength of evidence and statistical power by synthesizing all available related research (Riley, Higgins & Deeks, 2011). Meta-analysis of randomized controlled trials (RCTs) is recommended as the highest level of evidence in evidence-based practice (Puhan et al., 2014).

One important aim of meta-analyses of RCTs is to ascertain the causality between treatment and rare binary adverse events (AEs). Due to the low incidence of AEs or small sample size, the number of events tends to be low, even zero, in a single RCT. When zero events occur, the effect estimator (e.g., odds ratio, OR) and variance are generally undefined and make the synthesis of such studies challengeable. Traditional meta-analysis uses the inverse variance (IV) method to pool the effects of AEs, and a common modification is to add a continuity correction element (e.g., 0.5) for group sizes in the 2 × 2 table when there is no event in a single study (Bhaumik et al., 2012). The continuity correction method has been criticized for generating biased results, sometimes even reversing the direction of the effects, and was not recommended as the optimal choice (Rücker et al., 2009). Other superior methods, the Mantel-Haenszel (MH) and Yusuf-Peto (Peto) methods, were also developed to deal with rare binary AEs (Yusuf et al., 1985; Bradburn et al., 2010). These two methods used different point estimation and weighting schemes that allow a valid estimation of the effects and variance for single-zero studies (Rücker et al., 2009; Yusuf et al., 1985; Bradburn et al., 2010). Simulation studies suggested that MH and Peto showed less biased results than the continuity correction (Cheng et al., 2016; Sweeting, Sutton & Lambert, 2004). In the Cochrane handbook (version 5.2), the MH and Peto methods were listed as the standard methods for binary rare events (Chandler et al., 2017). However, when unbalanced trials exist, the Peto method was proved to be invalid due to substantial bias (Greenland & Salvan, 1990; Sharma, Gøtzsche & Kuss, 2017). When double-zero studies are introduced, none of the above methods can serve as solutions (Kuss, 2015).

More sophistic methods were then proposed to solve these problems. These include, for example, the Bayesian method, the Beta-binomial method, the Poisson-Gamma method, as well as the generalized linear mixed model for correlated outcomes (Sutton & Abrams, 2001; Cai, Parast & Ryan, 2010; Stijnen, Hamza & Ozdemir, 2010; Tian et al., 2009). The performance of these models for zero events were verified by large simulation studies (Sharma, Gøtzsche & Kuss, 2017). These methods were generally based on the aggregate data. But with the complex distribution and estimation procedure, they were seldom used in practice. Individual participant data (IPD) meta-analysis is seldom considered for dealing with rare binary events. This may partly be due the great difficulties often encountered in getting access for the raw data. As increasing numbers of trials shared data with the public, it becomes more feasible to use such types of data in meta-analysis for healthcare decision. Both “one-stage” and “two-stage” approaches are available, which have been described by Simmonds and Riley (Simmonds, Stewart & Stewart, 2015; Riley et al., 2015). For the one-stage approach, a multilevel model is employed by fitting individuals at the level-1 unit and studies at the level-2 unit within the whole dataset, to achieve pooling by a single logistic regression framework (Burke, Ensor & Riley, 2017). Indeed, the “one-stage” approach is a type of generalized linear mixed model, allowing the zero events in an individual study by “borrowing strength” from other parallel studies without zero events (Debray et al., 2013, 2015). The two-stage approach obtains the study-specific effects at the first stage and then pools the effects across studies using standard meta-analysis methods (i.e., IV, MH, and Peto) in the second stage (Scotti, Rea & Corrao, 2018). According to these features, the one-stage IPD meta-analysis based on multilevel logistic regression may merit consideration, because it applies a different synthesis procedure and variance handling method, and may achieve better parameter estimation, which is especially delicate for meta-analysis with zero events.

General logistic regression has been proven to be invalid with small samples (King & Zeng, 2001). This is because the maximum likelihood estimation would be systematically biased in such a situation. In spite of this, the method may still be reasonable under the rule of three, which indicates the “*number of trial subjects required in a trial to have a 95% chance of detecting an AE*” (Onakpoya, 2018). Under this rule, each trial is expected to have a 95% chance of detecting at least one AE case that may strengthen the power of logistic regression, although the event is still rare. For an AE with an incidence rate of 0.01 (i.e., one case in 100), a total of 300 subjects are required in a trial to ensure a 95% chance of detecting one case. There is currently no research that has applied this rule in logistic regression for meta-analysis of AE.

In this paper, we first introduce the use of one-stage IPD meta-analysis on rare binary AEs. We then illustrate the use of five classical methods (fixed and random effect IV, fixed and random effect MH, and Peto method) in a two-stage IPD framework. Finally, we compare the one-stage and the five classical methods in different simulation scenarios under the rule of three. The aim of the study was to test the feasibility of the one-stage approach for synthesizing binary AE data and to quantify its performance relative to the classical methods.

## Methods

### One-stage IPD meta-analysis for rare AE

The general concept of one-stage IPD meta-analysis was described in the introduction. For a binary outcome, it contains two exhaustive and mutually exclusive possible states: occurring or not occurring (Doi & Williams, 2013). The treatment-covariate interaction term is unnecessary under the assumption that all covariates were balanced in groups in RCTs as expected under randomization and assuming missing data does not depend on the covariates differently for some arms of the study. Then the multilevel variance component logistic regression (MVCL) model for one-stage IPD meta-analysis is:

$${y_{ij}} \sim {\rm{ln}}\left( {{{{p_{ij}}} \over {1 - {p_{ij}}}}} \right) = {{\rm{\alpha }}_j} + {\rm{\theta }}{x_{ij}} + {\rm{}}{e_{ij}}$$

$${e_{ij}}\sim N\left( {0,\sigma _e^2} \right)$$

where

$${y_{ij}}\sim {\rm{Bin}}\left( {{n_{ij}},{p_{ij}}} \right)$$

$${{\rm{\alpha }}_j} = {{\rm{\alpha }}_0} + {u_j}$$

$${u_j}\sim N\left( {0,{\rm{\sigma }}_u^2} \right),\quad{\rm{var}}\left( {{p_{ij}}} \right) = {p_{ij}}\left( {1 - {p_{ij}}} \right)/{n_{ij}}$$

Here *i* denotes individuals, *j* denotes studies included, and *y* is the binary outcome assigned as 1 (occurring) or 0 (not occurring), which obeys a binomial distribution of *n* (sample size) and *p* (probability). *p* denotes the estimated probability of a rare event occurring (*y* = 1). α_*j*_ is the intercept and consists of the fixed effect term α_*0*_ and the random term *u_j_*, with the variance σ_u_^2^ that refers to the between-study variance. θ is the fixed coefficient terms that refers to the occurring of AEs, and *x* is the matrix of covariates for the group of individuals (1 for the treatment group and 0 for the control group). *e_ij_* is the random error of the individual level, with the variance σ_e_^2^ that refers to the within-study variance.

Under this model, the pooled odds ratio (OR) for occurring AEs can be estimated directly in the multilevel regression model as:

$$\widehat {{\rm{OR}}} = {\rm{exp}}\left({\rm{\theta }} \right)$$

The estimation of θ in this model can be achieved by maximum likelihood or restricted maximum likelihood algorithms. The total variance of the model is the sum of between-study variance and within-study variance (σ_e_^2^ + σ_u_^2^). The magnitude of heterogeneity in the MVCL one-stage IPD meta-analysis can be expressed as:

$${I^2} = {{{\rm{\sigma }}_u^2} \over {{\rm{\sigma }}_e^2 + {\rm{\sigma }}_u^2}}$$

In the multilevel regression model, this term is also called the variance partition coefficient.

### Two-stage IPD meta-analysis for rare AEs

For standard two-stage IPD meta-analysis, the study-specific effect θ_*j*_ and the variance were obtained from each study using the regression model or 2 × 2 table.

$$y\sim {\rm{ln}}\left( {{p \over {1 - p}}} \right) = {\rm{\alpha }} + {\rm{\theta }}x + {\rm{}}e$$

$$e\sim N\left( {0,{\rm{\sigma }}_e^2} \right)$$

Then the five classical methods listed above were used to combine θ_*j*_ and the variance for rare binary AEs in the second stage. An assumption was made for the θ_*j*_ as:

$${{\rm{\theta }}_j} \sim N({{\hat{\rm\theta }},{\rm{\sigma }}_j^2 + {\tau ^2}} )$$

Here *τ*^2^ is the heterogeneity between studies and σ_j_^2^ is the within-study variance for θ_*j*_. When *τ*^2^ = 0, the model denotes a fixed effect model; otherwise, it is a random effect model. *W_j_* denotes the weight of each study, that:

$${W_j} = 1/({{\rm{\sigma }}_j^2 + {\tau ^2}})$$

For the sake of illustration, summary data of the 2 × 2 table of each study is introduced. We denote *a_j_*, *b_j_*, *c_j_*, and *d_j_* as the number of events and the number of non-events in the intervention and control groups. Under the IV method:

$${\rm{\sigma }}_{{\rm{IV}} - j}^2 = 1/{a_j} + 1/{b_j} + 1/{c_j} + 1/{d_j}$$

For the MH method, the variance is:

$${\rm{\sigma }}_{{\rm{MH}} - j}^2 = \left({{a_j} + {b_j} + {c_j} + {d_j}} \right)/{b_j}{c_j}$$

The pooled estimate of the IV and MH methods is calculated as:

$$\hat \theta = {{\left( {\sum {{W_j}} {\theta _j}} \right)} \over {\left( {\sum {{W_j}} } \right)}}$$

Unlike the IV and MH methods, the Peto method only contains a fixed effect model, with the pooled estimate as:

$${\hat{{\rm{\theta}}}_{{\rm{peto}}}} = {\rm{exp}}\left\{ {\left({{a_j} - E\left[ {{a_j}} \right]} \right)/{v_j}} \right\}$$

where *E*[*a_j_*] is the expected number of events in the intervention group and *v_j_* is the hypergeometric variance of *a_j_*. The calculation of *E*[*a_j_*] and *v_j_* have been described elsewhere (Harris, 2008).

The magnitude of the heterogeneity of between-study variance on the total variance is then:

$${I^2} = {{{\tau ^2}} \over {{{\rm{\sigma }}^2} + {\tau ^2}}};$$

Note that the within-study variance (σ^2^) is assumed to be equal across studies.

## Simulation

### Simulation parameters setting

The simulation was aimed at comparing the performance of the MVCL one-stage IPD meta-analysis method with five classical methods (based on the two-stage IPD meta-analysis method) for rare binary AEs under different simulation scenarios. In order to generate individual participants’ binary AE data, six key parameters were set, with different values to represent different simulation scenarios. The parameters were: incidence rate of rare AEs in the control group (*pc*); numbers of patients in control group in each individual study (*n*); adverse effect size (odds ratio, OR); between-study heterogeneity (tau, tau^2^ = *τ*^2^); number of studies in each meta-analysis (*m*); and the sample size ratio between the treatment and control group (*r*).

We first assigned *pc* with four values of 0.05, 0.01, 0.001, and 0.0001, which represented common, uncommon, rare, and very rare AE probabilities according to previous studies (Bhaumik et al., 2012; Rücker et al., 2009; Yusuf et al., 1985; Bradburn et al., 2010). The *n* can be calculated as $\left({{1 \over {pc}}} \right)*3$, based on the rule of three. Then, the sample sizes (*n*) of the control groups under the rule of three were 60 (*pc* = 0.05), 300 (*pc* = 0.01), 3,000 (*pc* = 0.001), and 30,000 (*pc* = 0.0001). In order to compare with the standard rule of three, we also considered two other situations to determining the individual study sample size—the rule of two, $\left({{1 \over {pc}}} \right)*2$, and the rule of one, ${\rm{}}\left( {{1 \over {pc}}} \right)*1$. The sample sizes (*n*) of the control group were then 40 (*pc* = 0.05), 200 (*pc* = 0.01), 2,000 (*pc* = 0.001), and 20,000 (*pc* = 0.0001) under the rule of two; and 20 (*pc* = 0.05), 100 (*pc* = 0.01), 1,000 (*pc* = 0.001), and 10,000 (*pc* = 0.0001) under the rule of one. The expected events in the control group were 3, 2, and 1 under the rule of three, rule of two, and rule of one, respectively. The group ratio (*r*) was set as 1:1, and then the incidence rate in the treatment group could be calculated by *pc* and OR.

We considered the effect sizes as four situations: no effect (OR = 1.0), mild harmful effect (OR = 1.25), moderate harmful effect (OR = 2), and large harmful effect (OR = 5). Theoretically, the beneficial effects would be consistent with the harmful effects on the simulation performance; therefore, we did not set scenarios on beneficial effects.

For *τ*, we considered three situations, as previous literature demonstrated that 0.1, 0.5, and 1.0 indicated mild, moderate and substantial between-study heterogeneity, respectively (Cheng et al., 2016).

For the number of included studies, Cheng et al. (2016) reviewed the previous literature and set *m* as 5, because that is the median number of published meta-analyses of RCTs. We agree with this assumption; however, we empirically added two scenarios (10 and 15) to cover more situations that are also commonly used in similar simulation studies (Doi et al., 2015a, 2015b). The key R code for simulations is presented in the Supplementary File.

### Simulation scenarios and statistics analysis

We compared the performance of the methods under different scenarios of sample size setting rules, effect sizes, between-study heterogeneity, and numbers of studies in each meta-analysis. This was achieved by setting two types of parameters: fixed and varied. For fixed parameters, a fixed value was assigned in each set of scenario; for varied parameters, different values were assigned according to the aim of comparison. For example, when comparing the performance of the methods under different sample size setting rules, we treated effect size, between-study heterogeneity, and numbers of studies as fixed parameters, and treated the sample sizes as varied parameters. Detailed simulation scenarios can be seen in Table 1.

**Table 1:** Parameter setup in different simulation scenarios.

Simulation scenarios	*pc*	*n*	OR	tau	*m*	*r*
Scenarios of rule of three/two/one						
1	0.05	60^△^	1.25	0.1	10	1
2	0.05	40	1.25	0.1	10	1
3	0.05	20	1.25	0.1	10	1
4	0.01	300^△^	1.25	0.1	10	1
5	0.01	200	1.25	0.1	10	1
6	0.01	100	1.25	0.1	10	1
7	0.001	3,000^△^	1.25	0.1	10	1
8	0.001	2,000	1.25	0.1	10	1
9	0.001	1,000	1.25	0.1	10	1
10	0.0001	30,000^△^	1.25	0.1	10	1
11	0.0001	20,000	1.25	0.1	10	1
12	0.0001	10,000	1.25	0.1	10	1
Scenarios of different adverse effects (OR)						
13–16	0.05	60^△^	1/1.25/2/5	0.1	10	1
17–20	0.01	300^△^	1/1.25/2/5	0.1	10	1
21–24	0.001	3,000^△^	1/1.25/2/5	0.1	10	1
25–28	0.0001	30,000^△^	1/1.25/2/5	0.1	10	1
Scenarios of different study heterogeneity (tau)						
29–31	0.05	60^△^	1.25	0.1/0.5/1	10	1
32–34	0.01	300^△^	1.25	0.1/0.5/1	10	1
35–37	0.001	3,000^△^	1.25	0.1/0.5/1	10	1
38–40	0.0001	30,000^△^	1.25	0.1/0.5/1	10	1
Scenarios of different number of studies in each meta-analysis (*m*)						
41–43	0.05	60^△^	1.25	0.1	5/10/15	1
44–46	0.01	300^△^	1.25	0.1	5/10/15	1
47–49	0.001	3,000^△^	1.25	0.1	5/10/15	1
50–52	0.0001	30,000^△^	1.25	0.1	5/10/15	1

**Notes:**

△ under the rule of three.

*pc*, incidence rate of rare AEs in control group.

*n*, number of patients in control group in each individual study.

OR, odds ratio, for measuring adverse effect size.

tau, between-study heterogeneity.

*m*, number of studies in each meta-analysis.

*r*, sample size ratio of between treatment and control group.

We compared the statistical performance of the MVCL one-stage method with the five classical meta-analytic methods in different scenarios. The following statistics were used to measure the performance: (1) the percentage bias, which is the percentage difference between the pooled OR and the true value; (2) the mean square error (MSE), which refers to the sample standard deviation of true and observed values; (3) the coverage probability, which indicates the proportion of times that the interval contains the true value; and (4) the average width of the 95% confidence interval, which is a reflection of the precision of effect sizes. Generally, smaller percentage bias, smaller MSE, larger coverage, and smaller average width of the 95% CI indicates better effect estimation.

A total of 52 simulation scenarios were defined according to the above conditions (Table 1). We simulated 1,000 data sets for each scenario, to ensure the accuracy of our simulation results. All of the simulations and statistics were conducted on R software (R Core Team, 2017); the MVCL one-stage IPD meta-analysis was based on the lme4 package and the five classical meta-analytic methods were based on the meta package.

## Results

### Performance under different sample size setting rules

Table 2 presents the performance of the six methods under three sample size setting rules with fixed values of effect size (OR = 1.25, mild effect), between-study heterogeneity (tau = 0.1, mild heterogeneity), and number of studies (*m* = 10). Our simulation study suggested that, under the rule of three, the MVCL one-stage IPD method had the lowest percentage of bias (<4%) of OR, regardless of the incidence rate. We then compared the performance of the six methods under the rule of two, in which the expected number of events in the control group decreased to two in a single trial. The bias increased for all of the six methods. However, the one-stage IPD method still generally had the lowest amount of bias, with the percentage of bias ranging from 4.9% to 7.2%. This was not the case under the rule of one when the incidence rate decreased to 0.001—the fixed effect IV, random effect IV, and random effect MH had the lowest biases; while when the incidence rate decreased to 0.0001, except for Peto method, the remaining methods tended to have similar biases (Table 2).

**Table 2:** The performance of the six methods under different sample size setting rules.

Methods in different incidence rate and sample sizes			OR = 1.25, tau = 0.1, *m* = 10											
			“One-stage” IPD		IV-*f*		IV-*r*		MH-*f*		MH-*r*		Peto	
Incidence rate	Sample size		Mean OR	Bias (%)	Mean OR	Bias (%)	Mean OR	Bias (%)	Mean OR	Bias (%)	Mean OR	Bias (%)	Mean OR	Bias (%)
*pc* = 0.05	Rule of three	60	1.29	3.15	1.43	14.31	1.46	17.18	1.43	14.33	1.42	13.48	1.42	13.87
	Rule of two	40	1.33	6.16	1.45	16.05	1.46	16.47	1.50	20.16	1.46	16.51	1.48	18.48
	Rule of one	20	1.42	13.71	1.53	22.32	1.53	22.39	1.55	24.23	1.53	22.40	1.72	37.82
*pc* = 0.01	Rule of three	300	1.30	3.76	1.40	12.24	1.41	12.61	1.45	15.67	1.41	12.67	1.40	11.91
	Rule of two	200	1.31	4.92	1.30	4.15	1.31	4.41	1.34	7.57	1.31	4.45	1.36	8.53
	Rule of one	100	1.40	11.72	1.40	11.94	1.40	12.05	1.43	14.37	1.40	12.06	1.59	27.49
*pc* = 0.001	Rule of three	3,000	1.29	3.13	1.34	6.89	1.34	7.09	1.37	9.74	1.34	7.14	1.35	7.80
	Rule of two	2,000	1.34	7.20	1.36	8.66	1.36	8.89	1.40	12.30	1.36	8.92	1.39	11.30
	Rule of one	1,000	1.41	13.00	1.32	5.21	1.31	5.20	1.34	7.16	1.31	5.20	1.47	17.56
*pc* = 0.0001	Rule of three	30,000	1.29	2.91	1.32	5.55	1.32	5.73	1.36	8.67	1.32	5.78	1.34	6.81
	Rule of two	20,000	1.34	7.17	1.38	10.02	1.38	10.53	1.43	14.07	1.38	10.57	1.44	15.42
	Rule of one	10,000	1.42	13.88	1.43	14.14	1.43	14.15	1.46	17.07	1.43	14.15	1.65	31.68

**Notes:**

Sample size was calculated by the three rules under different incidence rate.

*pc*, incidence rate of rare AEs in control group; OR, adverse effect size; tau, between-study heterogeneity; *m*, number of studies in each meta-analysis.

**Figure 1:**
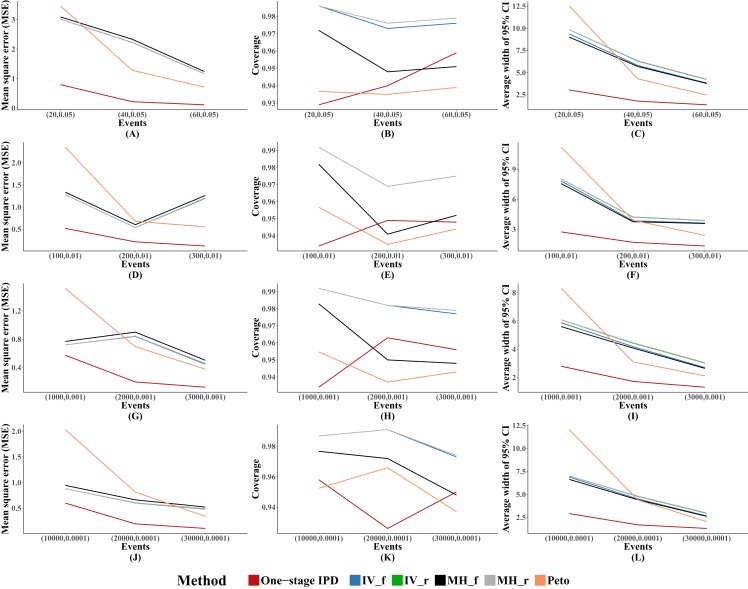
Mean square error (MSE), coverage, and width of 95% confidence interval with different sample size setting rules. This include “Rule of three,” “Rule of two,” and “Rule of one.” The solid lines indicate these statistics, and were distinguished by different colors for the six methods. The lines of fixed-effect model of inverse variance and MH methods were nearly coincide; similarly for the lines of random-effect model of inverse variance method and MH method. A–L indicate different scenarios of MSE, coverage, width of 95% confidence interval under different sample size setting rules.

For other performance indicators, generally the MVCL one-stage IPD method had the lowest MSE and the narrowest average width of 95% confidence intervals. However, the MVCL one-stage IPD method did not show better coverage probability compared to the other five methods (Fig. 1).

### Performance with different OR

Table 3 presents the performance of the six methods with different magnitudes of OR under the rule of three, with fixed between-study heterogeneity (tau = 0.1, mild heterogeneity) and numbers of studies (*m* = 10). The results of the simulation showed that the MVCL one-stage IPD meta-analysis generally gave the lowest percentage bias of the estimated OR and the bias remained very low (2.8–4.1%), regardless of the magnitude of the OR. The Peto method ranked the second lowest in bias in nine of 16 scenarios. However, when the incidence rate was 0.001 and 0.0001, the fixed and random effect IV methods and the random effect MH method performed better than the MVCL one-stage IPD when there were moderate and large effects (OR = 2 and OR = 5). We observed unstable results for the Peto method when there was a large effect (OR = 5) in different incidence rates.

**Table 3:** Magnitude of ORs on the influence of the performance of the six methods under the rule of three.

Different values of OR	Synthesis methods for rare AE under the rule of three, tau = 0.1, *m* = 10											
	“One-stage” IPD		IV-*f*		IV-*r*		MH-*f*		MH-*r*		Peto	
	Mean OR	Bias (%)	Mean OR	Bias (%)	Mean OR	Bias (%)	Mean OR	Bias (%)	Mean OR	Bias (%)	Mean OR	Bias (%)
*pc* = 0.05, *n* = 60												
OR = 1	1.03	3.06	1.15	15.03	1.15	14.90	1.16	15.76	1.15	14.90	1.14	13.75
OR = 1.25	1.29	3.15	1.42	13.87	1.43	14.31	1.46	17.18	1.43	14.33	1.42	13.48
OR = 2	2.06	3.21	2.28	14.02	2.31	15.58	2.43	21.40	2.31	15.71	2.24	12.09
OR = 5	5.17	3.32	6.86	37.25	7.04	40.79	7.42	48.49	7.06	41.13	5.62	12.36
*pc* = 0.01, *n* = 300												
OR = 1	1.03	3.37	1.14	13.81	1.14	14.12	1.15	15.19	1.14	14.15	1.13	12.89
OR = 1.25	1.30	3.76	1.40	12.24	1.41	12.62	1.45	15.67	1.41	12.67	1.40	11.91
OR = 2	2.07	3.35	2.14	7.04	2.16	7.95	2.30	14.78	2.16	8.08	2.12	6.08
OR = 5	5.21	4.11	5.32	6.49	5.40	7.91	5.90	18.01	5.42	8.31	4.17	−16.60
*pc* = 0.001, *n* = 3,000												
OR = 1	1.03	3.21	1.09	9.37	1.10	9.51	1.10	9.87	1.10	9.53	1.09	9.12
OR = 1.25	1.29	3.13	1.34	6.89	1.34	7.09	1.37	9.74	1.34	7.14	1.35	7.80
OR = 2	2.06	3.10	2.06	2.97	2.08	4.07	2.21	10.40	2.08	4.23	2.06	2.93
OR = 5	5.15	3.08	4.96	−0.73	5.04	0.85	5.51	10.23	5.06	1.27	3.88	−22.45
*pc* = 0.0001, *n* = 30,000												
OR = 1	1.03	2.75	1.08	7.90	1.08	7.74	1.09	8.64	108	7.76	1.08	7.82
OR = 1.25	1.29	2.91	1.32	5.55	1.32	5.73	1.36	8.67	1.32	5.78	1.34	6.81
OR = 2	2.06	2.79	2.03	1.29	2.04	1.98	2.16	8.18	2.04	2.08	2.03	1.62
OR = 5	5.15	3.04	4.85	−2.92	4.91	−1.71	5.35	7.04	4.93	−1.41	3.83	−23.44

**Notes:**

*pc*, incidence rate of rare AEs in control group; OR, odds ratio; tau, between-study heterogeneity; *m*, number of studies in each meta-analysis; *n*, number of patients in control group.

**Figure 2:**
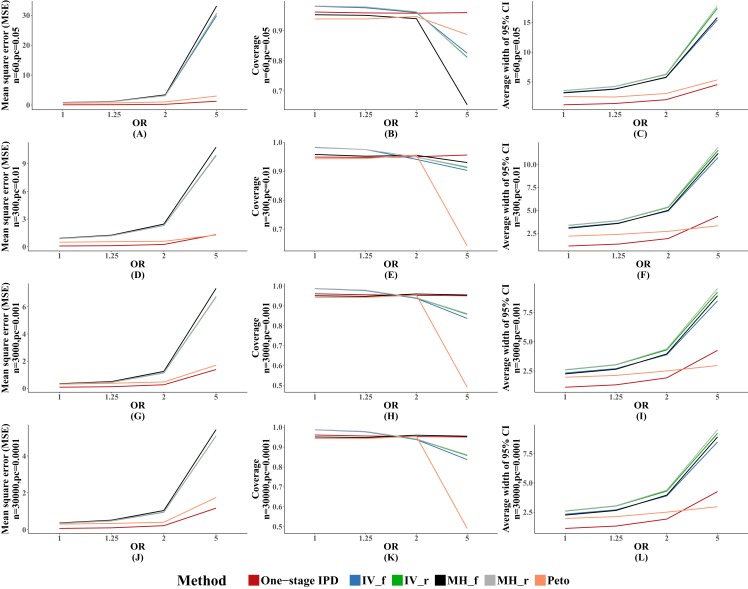
Mean square error (MSE), coverage, and width of 95% confidence interval with different ORs. The solid lines indicate these statistics, and were distinguished by different colors for the six methods. In some cells, the lines were coincided, especially for inverse variance and MH methods. A–L indicate different scenarios of MSE, coverage, width of 95% confidence interval under rule of three.

Figure 2 presents the average MSE, coverage probability, and average width of the 95% confidence intervals. The MVCL one-stage IPD method had the lowest MSE and the narrowest average width of 95% confidence intervals. All of the six methods had good coverage probabilities (>95%), while the IV method and random effect MH method tended to perform better than the MVCL one-stage IPD. Overall, with increasing OR there was an increasing trend for the MSE and the average width of the 95% confidence intervals, but the coverage remained stable.

### Performance with different heterogeneity

Table 4 presents the performance of the six methods with different between-study heterogeneity under the rule of three, with fixed effect size (OR = 1.25, mild effect) and numbers of studies (*m* = 10). The results showed that the MVCL one-stage IPD method performed well, with lower percentage bias than the other five methods when there was mild or moderate heterogeneity between studies. The Peto method performed better than the IV and MH methods in five of the 12 scenarios. However, when there was substantial between-study heterogeneity, all six methods presented an obviously large amount of bias such the most of the ORs were overestimated by more than 50%. For the IV, MH, and Peto methods, the percentage bias decreased as the heterogeneity decreased.

**Table 4:** The performance of the six methods on different magnitude of between-study heterogeneity under the rule of three.

Different values of tau	Synthesis methods for rare AE under the rule of three, OR = 1.25, *m* = 10											
	“One-stage” IPD		IV*-f*		IV-*r*		MH-*f*		MH-*r*		Peto	
	Mean OR	Bias (%)	Mean OR	Bias (%)	Mean OR	Bias (%)	Mean OR	Bias (%)	Mean OR	Bias (%)	Mean OR	Bias (%)
*pc* = 0.05, *n* = 60												
tau = 0.1	1.29	3.15	1.42	13.87	1.43	14.30	1.46	17.18	1.43	14.33	1.42	13.48
tau = 0.5	1.32	5.66	1.47	17.55	1.47	17.82	1.51	21.04	1.47	17.86	1.45	15.82
tau = 1.0	1.95	56.00	2.54	103.19	2.43	94.34	2.66	112.90	2.43	94.45	2.20	76.18
*pc* = 0.01, *n* = 300												
tau = 0.1	1.30	3.76	1.40	12.24	1.41	12.62	1.45	15.67	1.41	12.67	1.40	11.91
tau = 0.5	1.34	7.10	1.43	14.43	1.44	14.83	1.47	17.94	1.44	14.90	1.43	14.40
tau = 1.0	2.12	69.79	2.18	74.20	2.01	60.60	2.40	92.32	2.01	60.58	2.13	70.04
*pc* = 0.001, *n* = 3,000												
tau = 0.1	1.29	3.13	1.34	6.89	1.34	7.09	1.37	9.74	1.34	7.14	1.35	7.80
tau = 0.5	1.34	6.90	1.38	10.20	1.38	10.33	1.42	13.37	1.38	10.39	1.39	10.84
tau = 1.0	2.15	71.67	2.02	61.35	1.88	50.36	2.24	79.34	1.88	50.30	1.99	59.28
*pc* = 0.0001, *n* = 30,000												
tau = 0.1	1.29	2.91	1.32	5.55	1.32	5.73	1.36	8.67	1.32	5.78	1.34	6.81
tau = 0.5	1.33	6.45	1.34	7.44	1.34	7.38	1.38	10.53	1.34	7.41	1.36	8.95
tau = 1.0	2.14	71.58	1.91	52.91	1.77	41.89	2.15	71.75	1.77	41.84	1.95	55.66

**Notes:**

*pc*, incidence rate of rare AEs in control group; OR, odds ratio; tau, between-study heterogeneity; *m*, number of studies in each meta-analysis; *n*, number of patients in control group in each individual study.

**Figure 3:**
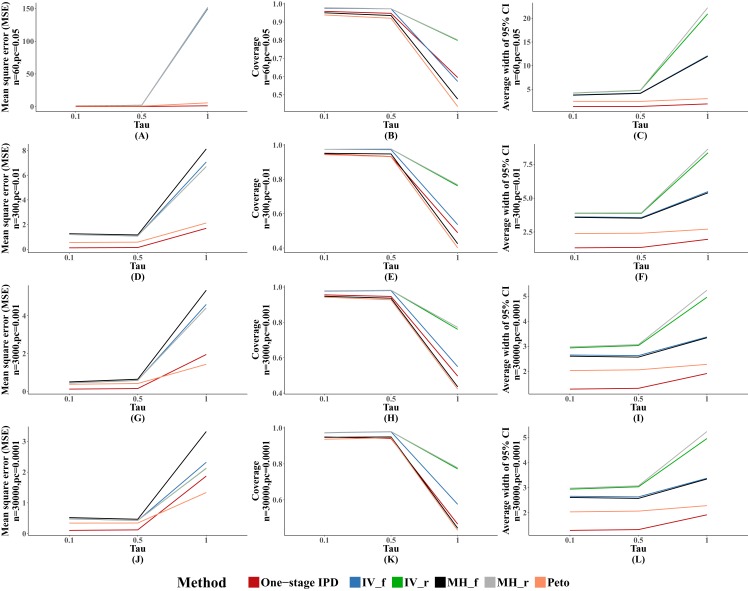
Mean square error (MSE), coverage, and width of 95% confidence interval with different between study heterogeneity (tau^2^). The solid lines indicate these statistics, and were distinguished by different colors for the six methods. In some cells, the lines were coincided, especially for inverse variance and MH methods. A–L indicate different scenarios of MSE, coverage, width of 95% confidence interval under rule of three.

We observed similar results for other performance indicators—the MVCL one-stage IPD method generated the lowest MSE and the narrowest average width of the 95% confidence intervals, and the IV and random effect MH methods tended to cover more true values. The MSE and the average width of the confidence intervals increased when higher between-study heterogeneity was present (Fig. 3).

### Performance with different numbers of studies

Table 5 presents the comparison of the MVCL one-stage IPD method with the other five methods with different numbers of studies included under the rule of three, with fixed effect size (OR = 1.25, mild effect) and between-study heterogeneity (tau = 0.1, mild heterogeneity). Our simulation data showed that the MVCL one-stage IPD method consistently presented lower bias than the other five methods. Furthermore, when the number of included studies increased, the bias decreased with all of the methods. When the number of included studies was 15, the bias remained very low level with all of the methods.

**Table 5:** The performance of the six methods on different number of studies included in a meta-analysis under the rule of three.

Difference values of *m*	Synthesis methods for rare AE under the rule of three, OR = 1.25, tau = 0.1											
	“One-stage” IPD		IV-*f*		IV-*r*		MH-*f*		MH-*r*		Peto	
	Mean OR	Bias (%)	Mean OR	Bias (%)	Mean OR	Bias (%)	Mean OR	Bias (%)	Mean OR	Bias (%)	Mean OR	Bias (%)
*pc* = 0.05, *n* = 60												
*m* = 5	1.36	8.64	1.43	14.34	1.44	15.15	1.47	17.88	1.44	15.25	1.46	16.52
*m* = 10	1.29	3.15	1.42	13.87	1.43	14.31	1.46	17.18	1.43	14.33	1.42	13.48
*m* = 15	1.28	2.64	1.37	9.68	1.37	10.14	1.41	13.13	1.38	10.18	1.39	10.95
*pc* = 0.01, *n* = 300												
*m* = 5	1.38	10.03	1.48	18.47	1.49	19.02	1.52	21.80	1.49	19.10	1.44	15.59
*m* = 10	1.30	3.76	1.40	12.24	1.41	12.62	1.45	15.67	1.41	12.67	1.40	11.91
*m* = 15	1.28	2.02	1.29	3.27	1.29	3.37	1.33	6.48	1.29	3.38	1.31	5.17
*pc* = 0.001, *n* = 3,000												
*m* = 5	1.33	6.23	1.40	11.74	1.40	12.31	1.43	14.62	1.40	12.36	1.38	10.43
*m* = 10	1.29	3.13	1.34	6.89	1.34	7.09	1.37	9.74	1.34	7.14	1.35	7.80
*m* = 15	1.27	1.63	1.31	5.04	1.32	5.20	1.35	8.11	1.32	5.23	1.33	6.04
*pc* = 0.0001, *n* = 30,000												
*m* = 5	1.33	6.00	1.34	7.45	1.35	7.66	1.38	10.16	1.35	7.69	1.38	10.16
*m* = 10	1.29	2.91	1.32	5.55	1.32	5.73	1.36	8.67	1.32	5.78	1.34	6.81
*m* = 15	1.28	2.12	1.34	7.00	1.34	7.38	1.39	10.91	1.34	7.44	1.35	8.23

**Notes:**

*pc*, incidence rate of rare AEs in control group; OR, odds ratio; tau, between-study heterogeneity; *m*, number of studies in each meta-analysis; *n*, number of patients in control group in each individual study.

**Figure 4:**
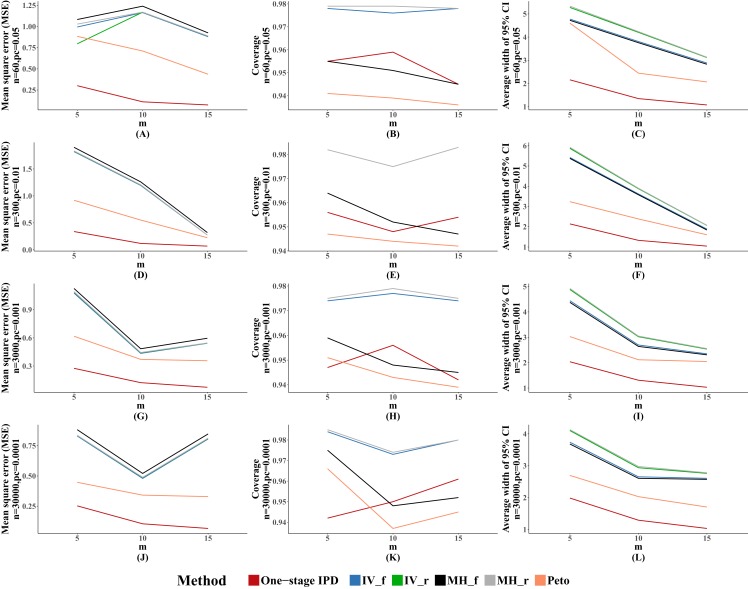
Mean square error (MSE), coverage, and width of 95% confidence interval with different number of studies included (*m*). The solid lines indicate these statistics, and were distinguished by different colors for the six methods. In some cells, the lines were coincided, especially for inverse variance and MH methods. A–L indicate different scenarios of MSE, coverage, width of 95% confidence interval under rule of three.

The MSE and average width of confidence intervals of the MVCL one-stage IPD method remained the best of the methods, and the number of studies was inversely associated with the magnitude of MSE and the average width of the 95% confidence intervals. The coverage of the MVCL one-stage method generally performed less well than the IV method and the random effect MH method (Fig. 4).

## Discussion

In this simulation study, we investigated the performance of MVCL one-stage IPD meta-analysis relative to the IV, MH, and Peto methods, for rare events based on IPD. Our results found that, under the rule of three, the one-stage IPD method had the lowest bias in most situations, and that the bias remained at a very low level when the between-study heterogeneity was not substantial. When there was substantial between-study heterogeneity, all of the methods generated a large amount of bias.

According to our simulation, the MVCL one-stage IPD method generally had the lowest MSE and the narrowest average width of the 95% confidence intervals. However, it did not show better coverage probability than the other five methods. This finding is consistent with the study by Debray et al. (2013), which compared the performance of the one-stage IPD method with the two-stage method using an empirical dataset. In the study, Debray et al. (2013) concluded that the one-stage approach generally does not converge. This is probably related to the average width of the confidence intervals of the one-stage approach. A narrow confidence interval means higher precision while usually sacrificing probability to capture the true value. However, in this simulation, the one-stage approach was verified to generate an almost identical estimation to the true value.

We further observed that, among the IV, MH, and Peto methods, the Peto method generally performed best under the rule of three. Moreover, the fixed effect MH had the highest amount of bias across all methods. This finding was consistent with the simulation by Cheng et al. (2016), who compared the performance of the IV, MH, and Peto methods in a scenario of a high prevalence of zero events. They found that the Peto method gave the lowest estimation bias when excluding both-arm-zero trials. However, in the current simulation, we observed unstable results for the Peto method: in several situations, the fixed IV and the random MH method performed better than the Peto method, though their estimations were similar.

In this simulation study, we observed that 41 of the 52 scenarios reported an overestimation of the ORs. The MVCL one-stage IPD method presented a mild overestimation (about 3%) of the true value when the between-study heterogeneity was acceptable. Under the same situation, the other five methods overestimated the true value by 10–20%. However, when there was substantial heterogeneity, all six methods presented an obviously large amount of bias and the effect sizes (ORs) were mostly overestimated by more than 50%. This finding suggests that the results should be interpreted more conservatively when there is substantial heterogeneity.

An interesting phenomenon in the results is that, for the MVCL one-stage IPD method under the rule of three, the percentage bias remained relatively stable with different levels of incidence rates. However, for the IV, MH, and Peto methods, there was an inverse association between the incidence rate and the percentage bias: when the incidence rate decreased, the estimations under the IV, MH, and Peto methods became more accurate. Moreover, when there was a sufficient number of included studies (e.g., *m* = 15), the bias tended to be diminished in all of the methods.

To the best of our knowledge, this is the first study to use the MVCL one-stage IPD meta-analytic method to deal with rare binary AEs. We used comprehensive simulation scenarios to compare the performance of the MVCL one-stage IPD method with classical methods under the rule of three. In this simulation study, we verified the value and feasibility of the MVCL one-stage IPD method on binary AEs, and therefore recommend that it be considered in further meta-analysis of AEs. Nevertheless, there were several limitations of the current method. First, the MVCL “one-stage” IPD method performed well under the rule of three and rule of two, but not under the rule of one. This required two AEs to be expected to occur in the control group of included studies. However, this restriction could be solved by exact logistic regression, Firth logistic regression, and rare event logistic regression (King & Zeng, 2001; Mehta & Patel, 2010). Unfortunately, there is no available package for modelling multilevel regressions for these methods. We will compare these new methods to the current one in a further study when there is an appropriate solution.

The second limitation is that, for the current one-stage IPD model, we fitted a multilevel variance component model (MVCL) but not a multilevel random coefficient model (MRCL). This is because the aim of the current study was to test the feasibility of the one-stage approach; we will compare the MVCL and MRCL models in a future simulation study. Moreover, the current method requires individual participants’ data, while the process to get access to IPD is costly and time-consuming.

## Conclusion

In conclusion, the MVCL one-stage IPD meta-analysis is a useful and superior method for handling binary rare events under the rule of three. It appears to produce more accurate parameter estimation and more precise interval estimation than traditional methods for rare events. It is recommended that future meta-analyses take account of the one-stage IPD method.

## Supplemental Information

10.7717/peerj.6295/supp-1 Supplemental Information 1 Supplimentary file. Simulation code for the study.

## Additional Information and Declarations

### Data Availability

The following information was supplied regarding data availability: The raw code is available as a Supplementary File.
